# Rationale and design of a double-blinded, randomized placebo-controlled trial of 40 Hz light neurostimulation therapy for depression (FELIX)

**DOI:** 10.1080/07853890.2024.2354852

**Published:** 2024-05-20

**Authors:** Laura Sakalauskaitė, Luna S. Hansen, Julie Margrethe Dubois, Malina Ploug Larsen, Gustavo Miguel Feijóo, Marcus S. Carstensen, Kamilla Woznica Miskowiak, Mai Nguyen, Line Katrine Harder Clemmensen, Paul Michael Petersen, Klaus Martiny

**Affiliations:** a New Interventions in Depression Group (NID-Group), Copenhagen Affective Disorder Research Centre (CADIC), Psychiatric Centre Copenhagen, Copenhagen University Hospital, Copenhagen, Denmark; b Department of Electrical and Photonics Engineering, The Technical University of Denmark; c OptoCeutics ApS, Lyngby, Denmark; d Neurocognition and Emotion in Affective Disorders (NEAD) Group, Copenhagen Affective Disorders Research Center (CADIC), Psychiatric Centre Copenhagen, Copenhagen University Hospital, Copenhagen, Denmark; e Department of Applied Mathematics and Computer Science, The Technical University of Denmark, Lyngby, Denmark

**Keywords:** Light stimulation, major depressive disorder, visual flicker, 40 Hz stimulation, gamma brainwaves, cognition

## Abstract

**Background:**

Major depressive disorder (MDD) is a debilitating condition that affects more than 300 million people worldwide. Current treatments are based on a trial-and-error approach, and reliable biomarkers are needed for more informed and personalized treatment solutions. One of the potential biomarkers, gamma-frequency (30–80 Hz) brainwaves, are hypothesized to originate from the excitatory-inhibitory interaction between the pyramidal cells and interneurons. The imbalance between this interaction is described as a crucial pathological mechanism in neuropsychiatric conditions, including MDD, and the modulation of this pathological interaction has been investigated as a potential target. Previous studies attempted to induce gamma activity in the brain using rhythmic light and sound stimuli (GENUS – Gamma Entrainment Using Sensory stimuli) that resulted in neuroprotective effects in Alzheimer’s disease (AD) patients and animal models. Here, we investigate the antidepressant, cognitive, and electrophysiological effects of the novel light therapy approach using 40 Hz masked flickering light for patients diagnosed with MDD.

**Methods and design:**

Sixty patients with a current diagnosis of a major depressive episode will be enrolled in a randomized, double-blinded, placebo-controlled trial. The active treatment group will receive 40 Hz masked flickering light stimulation while the control group will receive continuous light matched in color temperature and brightness. Patients in both groups will get daily light treatment in their own homes and will attend four follow-up visits to assess the symptoms of depression, including depression severity measured by Hamilton Depression Rating Scale (HAM-D_17_), cognitive function, quality of life and sleep, and electroencephalographic changes. The primary endpoint is the mean change from baseline to week 6 in depression severity (HAM-D_6_ subscale) between the groups.

## Introduction

Major Depressive Disorder (MDD) is a common and serious medical illness characterized by often recurrent depressive episodes. While antidepressants and psychotherapy can be effective in managing the symptoms of depression, they fail to achieve remission in approximately one-third of individuals [1].

Currently, bright light therapy (BLT) is used for the treatment of seasonal depression. Although it has shown some effectiveness in non-seasonal MDD, the quality of the evidence is poor and a substantial rate of patients do not reach remission [2,3]. The reasons for this difference in effectiveness are not entirely clear, but BLT is known to affect mainly the circadian system by stimulating specific cells in the retina, suppressing melatonin production, and increasing serotonin levels [4]. Although the circadian system has profound effects on mood and general well-being, MDD is a more complex disorder with various genetic and environmental factors, brain regions, and neural networks contributing to its pathophysiology [5,6].

Depression affects multiple large-scale functional networks in the brain, with measures such as resting-state functional connectivity within the default mode network distinguishing depressed patients from healthy controls [7–10] or predicting response to specific antidepressant medications [11,12]. To address the problem of ineffective pharmacotherapy, various brain stimulation approaches, such as electroconvulsive therapy, transcranial magnetic or electrical stimulation, pulsed electromagnetic field therapy and deep brain stimulation, have been utilized or tested [13–16]. It is hypothesized that these therapies improve the symptoms of depression by modulating the brain at the network level.

Brain network activity could be modulated by various external stimuli, either magnetic, electrical or even auditory and visual [17]. For example, transcranial alternating current stimulation at 10 Hz has been investigated for the treatment of depression and hypothesized to improve clinical symptoms by renormalizing alpha oscillations in the left dorsolateral prefrontal cortex [18]. The brain can respond and synchronize to external sensory stimuli. Studies have shown that light flicker stimulus at specific frequencies can entrain the same frequency waves beyond the visual pathway in the hippocampus and prefrontal cortex [19–21]. Targeted activation of these regions could be beneficial for the integrity of structure and function of the underlying cells. For example, the hippocampus is a critical brain region responsible for memory and emotions [22,23], and the shrinking of the hippocampus is described in people with recurrent and poorly treated depressive episodes. A study using 3 months of 40 Hz daily stimulation on healthy elderly and AD patients has demonstrated lesser ventricular and hippocampal atrophy and better performance on face-name association delayed recall test [24]. This could indicate potential benefits and targeted approaches in patients with recurrent and difficult to treat depression.

In addition, intrinsic gamma activity measured by electroencephalography (EEG) or magnetoencephalography (MEG) is associated with higher cognitive functioning, information processing, and working memory [23,25]. Cognitive deficits occurring with depression have an essential role in treatment response and prognosis, and targeting the cognitive domain in depression could be a promising therapeutic approach [26,27]. Sensory stimulation with 40 Hz has not only been able to entrain gamma activity but increased the expression of brain-derived neurotrophic factor in mice [28], cleared up amyloid and tau proteins involved in Alzheimer’s disease (AD) rodent models [19,29–31], and improved mood, sleep, and daily functionality in patients with AD [31–33].

Even though the mechanisms behind gamma entrainment using sensory stimuli (GENUS) are not fully understood, considering the signs of hippocampal involvement and neuroprotective and cognitive effects associated with 40 Hz light stimulation, we want to investigate how this stimulation approach would affect brain network dysfunction and symptoms of depression. We hypothesize that daily 1-hour 40 Hz masked flickering light stimulation for 6 weeks can improve symptoms such as depressed mood, cognitive impairment, and disrupted circadian rhythms.

We aim to investigate the isolated effects of 40 Hz masked light by having a continuous color-temperature and brightness-matched light as a placebo comparator.

## Patients and methods

### Study participants

The study will recruit, in all, 60 males and non-pregnant females ages 18–75 with unipolar, non-psychotic MDD (according to DSM-5 criteria) who have a Major Depression Inventory (MDI) score of 21 or above and with a low risk of suicide (based on the Hamilton Depression Rating Scale item 3 suicidality question). Participants will be only included if they are on stable antidepressant medication and/or psychotherapy for at least two weeks before starting the trial and have no planned change of the medication regime for the 8 weeks study period. Only participants who are willing to comply with the scheduled plan and can use the device for one hour per day for 6 weeks will be included in the study.

The Mini International Neuropsychiatric Interview (M.I.N.I.) [34] will be administered to confirm the diagnosis of major depressive disorder and exclude co-existing conditions mentioned in the study protocol’s exclusion criteria. Scales regarding depression symptoms, suicidal ideation, sleep patterns, mania symptoms, and side effects will be recorded during the study visits.

Eligible patients will be recruited through outpatient clinics at Psychiatric Centre Copenhagen. Patients diagnosed with a major depressive episode will be recruited *via* their contact person at the outpatient mental health services. The contact person will ask patients if they are interested in being informed about the trial. If patients agree, a meeting between the participant and a study investigator will be scheduled. Patients will be assessed for eligibility based on inclusion and exclusion criteria predefined in the protocol. Informed consent will be obtained before the start of trial activities.

Patients with any history of seizures, photosensitive migraines, or eye disorders with light sensitivity will be excluded. The study aims to investigate the effects on unipolar, non-psychotic depression; therefore, patients with a known history of bipolar disorder or current psychotic symptoms will not be included in the investigation. The summary of all inclusion and exclusion criteria is provided in (Table 1).

**Table 1.** List of inclusion and exclusion criteria.

Inclusion	Exclusion
18–75 years Diagnosis of major depressive episode according to DSM-5 MDI score > 21 at screening Stable antidepressant medication and/or psychotherapy for at least two weeks before starting the trial. No planned change of antidepressant medication for the 8-weeks study period Willingness to comply with scheduled plan and using the device for 1 h per day for 42 days. Understanding the oral and written study information and willing to sign an informed consent.	History of photosensitive migraines and/or epileptic seizures Known eye disorders that might be sensitive to light treatment. Diagnosis of bipolar disorder according to DSM-5 criteria Suicidal ideation corresponding to a score of 2 or more on the HAM-D_17_ scale item 3 or if patient or investigator is uncertain of the degree of suicidal risk. Current psychotic symptoms. However, subjects with a prior psychotic depression or subjects with an actual psychotic depression episode that at time of informed consent no longer fulfils the psychosis criteria are allowed to participate. Current drug or alcohol dependence based on their medical records or the M.I.N.I. interview. Diagnosis of borderline personality disorder according to DSM-5 criteria Being enrolled in another investigational treatment study. Progressive neurodegenerative or neoplastic disease. Inability to understand the study procedures or handling of the NSS device. Pregnancy at time of inclusion or unsafe contraception in women of fertile age

Patients will receive standard of care therapy for their depression during the clinical trial. Only patients with any unforeseen changes in medication will be excluded from the study.

The study protocol was designed according to the Standard Protocol Items: Recommendations for Interventional Trials (SPIRIT) guidelines.

## Study design and interventions

The study is a randomized, double-blinded, placebo-controlled, single-center clinical trial investigating the effects of 40 Hz brain stimulation. Patients are randomly assigned to one of two groups: 40 Hz masked flickering white light (active) and continuous non-flickering white light matched in color temperature and brightness (placebo). This is a double-blind study, so neither the participant nor the researcher will know which treatment the participant is receiving. Both kinds of light appear as white light, so the normal white light serves as a well-matched placebo. As a precaution, the investigators doing the assessments will not see the devices turned on but will instruct the trial participants on correct device use during the baseline meeting. Each participant is handed out a user manual with detailed instructions on how to handle the light therapy and recommended daily usage times. Participants will receive in-person instructions and will be additionally contacted *via* phone to ensure a smooth process and proper implementation of the light therapy.

Participants take light therapy at home; however, the variability in the specific implementation details of the treatment, particularly among subjects with poor adherence, poses potential challenges to the trial’s overall outcome. To mitigate these concerns and enhance the integrity of the study, adherence to the treatment protocol in both groups is monitored using an eye-tracking system. This system documents each participant’s active usage time, ensuring a comprehensive assessment of treatment adherence.

A treatment blinding test will be administered to the participants at day 42. The treatment blinding test assesses the participant’s awareness of which treatment group, he/she was allocated to for assessment of blinding efficacy. In addition, an evaluation of participants’ expectations regarding the effectiveness of the treatment will be systematically conducted at the initial visit, serving as a baseline measure.

An independent investigator will program 30 active treatment and 30 placebo devices in total assigned with labels A and B to the device identification numbers. The statistician creates unequal randomization blocks (e.g. ABBA, BAABBA) where he assigns a patient number (e.g. FELIX01, FELIX02) to the treatment group (A or B) and then the treatment group to a random device (corresponding to the group). The block size is blinded to investigators.

**Figure 1.**
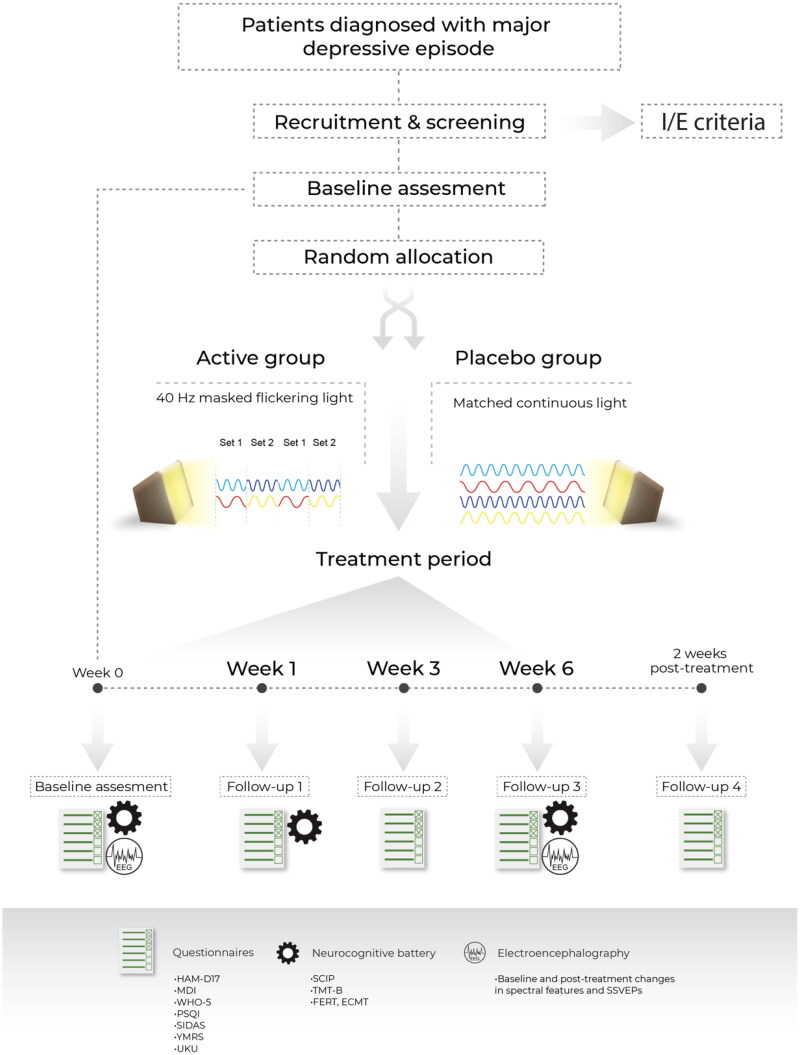
Visual representation of study design and follow-up visits. HAM-D_17_: Hamilton Depression Rating Scale 17 item version; MDI: Major Depression Inventory; WHO-5 World Health Organization Quality of Life Index; PSQI: Pittsburg Sleep Quality Index; SIDAS: Suicidal Ideation Assessment Scale; YMRS: Young Mania Rating Scale; UKU: The UKU Side Effect Rating Scale; SCIP: The Screen for Cognitive Impairment in Psychiatry. TMT-B: Trail Making Test Part B; FERT-Facial Emotion Recognition Task; ECMT: Emotional Categorization and Memory Test; SSVEPs: Steady State Visually Evoked Potentials.

The treatment period will last six weeks, and patients will come to their final follow-up visit at week 8 to assess any lasting antidepressant effects of light treatment. The study procedures include five visits with assessments using validated clinical scales administered by trained healthcare professionals, self-administered symptom, and quality of life scales. Additionally, neurocognitive evaluation and EEG procedures will be conducted (Figure 1).

Patients are free to withdraw from the trial at any time. Drop out criteria include hospitalisation due to a somatic illness, admission to a psychiatric ward for any length of time, side effects at a level unacceptable to the subject, worsening of depression such that it would be unethical not to try other treatments, the patient or the patient’s healthcare provider changes the dose of medication affecting the depression, onset of mania assessed according to the YMRS with a score of 7 or above or fulfilling the diagnostic DSM-5 criteria for a current hypomanic or manic episode, and patients reports conditions treatment that interfere with blinding. Patients who drop out from the trial continue their treatment and follow-up care at Psychiatric Centre Copenhagen.

A GCP monitor (GCP-Unit, University of Copenhagen, Frederiksberg Hospital) has been assigned to the study and will follow the clinical trial, randomization, blinding, and data collection procedures.

### Invisible spectral flicker and light characteristics

**Figure 2.**
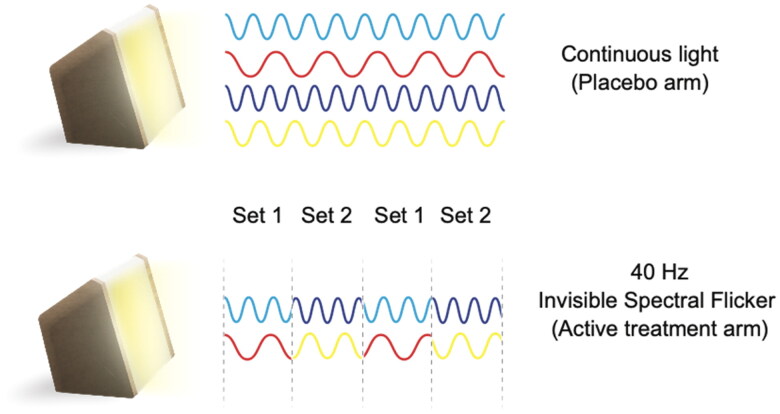
Representation of light paradigms and the design of invisible spectral flicker. Set 1: Cyan and red, set 2: blue and yellow.

A light device that delivers a 40 Hz light stimulus (OptoCeutics) will be used in the study. Unlike stroboscopic light, the device introduces the flickering stimulus by alternating between two color combinations (Figure 2). Alternating between two white light sets makes the flickering almost imperceivable to the human eye, therefore reducing discomfort related to stroboscopic or flashing light.

A traditional bright light therapy approach introduces app. 10k lux at the distance of 30 cm for 30 min or 2.5 k lux at 30 cm for 1 h [4]. The light used for the study emits <1500 lux at 30 cm and is not intended for the treatment of seasonal affective disorder (SAE) or as an alternative for bright light therapy. However, the light uses a wide spectrum of colors, including shorter wavelength cyan and navy blue, which are known to have a greater effect on the circadian system.

Melanopic Equivalent Daylight illuminance (mEDI) is an essential measure in light therapy studies and for quantifying the biological impact of light on the human visual system. The measure represents the melanopsin-weighted stimulus received by the intrinsically photosensitive retinal ganglion cells (ipRGCs) in response to light exposure. These specialized cells are responsible for conveying non-visual light information to various parts of the brain, including the suprachiasmatic nucleus (SCN), which regulates the body’s circadian rhythms. The ipRGCs are particularly sensitive to short-wavelength (blue) light and play a crucial role in regulating various physiological processes, such as the sleep-wake cycle, alertness, mood, and hormone production [35–37].

Measuring the mEDI in our study is essential as it controls for the potential effects of light on the circadian system and associated physiological responses, such as improved mood or quality of sleep. Both white light paradigms used in the study (masked flickering light and placebo continuous light) should have the same effects on the circadian system to compare the isolated effects of 40 Hz stimulation. [38] The light characteristics of both treatment conditions are described in (Table 2).

**Table 2.** Light matching in two different treatment conditions.

Group	CCT [K]	u’	v’	Radiant intensity [W/sr]	α-opic EDI metrics [lx]				
					Melanopic	Cyanopic	Chloropic	Erythropic	Rhodopic
Active	3.186	0.2478	0.5061	0.3522	**292**	180	325	383	308
Placebo	3.197	0.2479	0.5046	0.3624	**300**	186	327	384	315

CCT: Correlated Color Temperature (Kelvin), u’ and v’ are chromaticity coordinates used to describe the color of light. EDI: Equivalent Daylight Illuminance. Field of view/angle of the measurement device is 2°. Measurements are made at 50 cm distance.

## Study assessments and endpoints

Study assessments include questionnaires pertaining to the symptoms of depression, and include the assessment of depression severity, suicidal ideation, cognition, sleep duration and quality, general quality of life and mania. Summary of study evaluations and endpoints can be found in (Table 3).

**Table 3.** Summary of study endpoints.

	Endpoints	Description
Primary	Hamilton Depression Rating Scale 6 (HAM-D_6_) Time points of measurement: baseline, day 7, day 21, day 42, and day 56. The primary endpoint is the mean difference in scores between treatments at baseline and day 42.	The scale is designed to rate the severity of depression in patients by a healthcare professional. The assessment scale contains 6 items pertaining to the symptoms of depression experienced over the last three days. The score ranges from 0 to 24 [0–4] = no depression [5–6] = subclinical depression [7–8] = mild depression [9–11] = moderate depression [12–22] = moderate-severe depression A 2–3-point change in score is considered a minimum clinically important difference (MID) [39,50].
Secondary	Major Depression Inventory (MDI) Time points of measurement: baseline, day 7, day 21, day 42, and day 56	MDI is a depression self-assessment questionnaire. It consists of the 10 ICD-10 symptoms of depression. The sum of 10 questions indicates the degree of depression. The score ranges from 0 to 50 <21 = normal [21 − 25] = mild depression [26–30] = moderate depression ≥ 31 = severe depression [41]
	Neurocognitive battery Time points of measurement: baseline, day 7, and day 42	Assessing attention and recognition of emotional facial expressions using Facial Expression Recognition Test (FERT) and self-referent memory for emotional words using the Emotional Categorization and Memory test (ECMT). Non-emotional cognition is investigated with the Screen for Cognitive Impairment in Psychiatry (SCIP) and the Trail Making Test B (TMT-B) [51,52].
	Sleep diary covering last 7 days. Time points of measurement: continuous, by subjects themselves	Average last 7 days’ sleep parameters: Duration, falling asleep and waking up times, sleep quality, daytime sleep.
	Pittsburg Sleep Quality Index (PSQI) Time points of measurement: Baseline, and day 42	Seven component scores are derived, each scored 0 (no difficulty) to 3 (severe difficulty). The component scores are summed to produce a global score (range 0–21). Higher scores indicate worse sleep quality [53].
	WHO quality of life index (WHO-5) Time points of measurement: Baseline and day 21, day 42, and day 56	The score ranges from 0 to 25, with 0 representing the worst possible and 25 representing the best possible quality of life. To obtain a percentage score ranging from 0 to 100, the raw score is multiplied by 4. A percentage score of 0 represents the worst possible, whereas 100 represents the best possible quality of life. A 10% difference indicates a significant change [54].
Safety	The UKU Side Effects Scale Adverse Event (AE) Report Form The safety endpoint is the presence or absence of any adverse events during the treatment period.	All device-related adverse events will be reported and analyzed throughout the treatment and follow-up periods [55].
	Suicidal Ideation Attributes Scale (SIDAS) Time points of measurement: baseline, day 7, day 21, day 42, and day 56	The scale consists of 5 items: frequency, controllability, closeness to attempt, level of distress, and impact on daily functioning. Items are measured on a 10-point scale. The total score ranges from 0 to 50 (50 = worst). A cut-off of 21 may indicate a high risk of suicidal behavior [56].
	Young Mania Rating Scale (YMRS) Time points of measurement: Baseline, day 7, day 21, day 42, and day 56	The Scale assesses the manic symptoms that the light therapy could induce. The scale has 11 items and is based on the patient’s subjective report on his/her clinical condition over the last 48 h. Core mania symptoms include elevated mood, increased motor activity, irritability, and aggressive behavior. The Young Mania Rating Scale total score ranges from 0 to 60, where higher scores indicate more severe mania [57].
Adherence	The eye-tracking function of the device	Average device usage in minutes per day will be calculated. High adherence is considered 70% of the recommended duration of 1-h treatment per day.

### Assessment of depression severity

Hamilton Depression Rating 17 item (HAM-D_17_) and its subscale (HAM-D_6_) – is an interviewer-administered scale for rating the severity of depression. The HAM-D_6_ scale is a subscale of the HAM-D_17_ that has shown superior psychometric properties [39,40] and includes 6 items from the original scale, including depressed mood, work and activities, somatic symptoms, psychic anxiety, guilt, and psychomotor retardation. The HAM-D_6_ scale is rated for the last 3 days. The score range for the full scale is 0–52 and from 0 to 24 on the subscale, and the subscale scores between 9 and 11 are considered moderate depression, and scores above 11 – moderate-severe depression.

Major depression inventory (MDI) will be used as a self-report measure of depression severity. Patients will evaluate their depression symptoms at baseline and during every follow-up visit. The inclusion criteria for this study is an MDI score of 21 or above [41].

### Cognitive evaluation

It is hypothesized that antidepressant drugs reverse negative bias in the cognitive processing of emotional information [42]. This effect has been shown to occur early in the course of antidepressant drug treatment and predicts treatment response [43]. Therefore, in this RCT, we investigate for the first time whether 40 Hz masked flickering light therapy produces a similar early shift in the cognitive response to emotional information in depression and whether this early change is related to treatment efficacy. We will also investigate whether 40 Hz mask flickering light improves non-emotional cognitive functions in parallel with its effects on depressive symptoms.

The neurocognitive test battery assesses emotional and non-emotional cognition. The recognition of emotional facial expressions will be assessed using the Facial Expression Recognition Test (FERT) and self-referent memory for emotional words using the Emotional Categorization and Memory Test (ECMT) [44]. Non-emotional cognition will be investigated with the Screen for Cognitive Impairment in Psychiatry (SCIP) [45,46] and the Trail Making Test A and B (TMT-A and TMT-B) [47]. SCIP is a well-evaluated screening instrument for examining cognitive performance in psychiatric patients and includes list learning, consonant repetition, verbal fluency, delayed list learning, and visuomotor tracking tests [48]. TMT is a short additional test that evaluates visual attention (part A) and attention switching (part B; executive function), respectively.

### Evaluating changes in sleep patterns

Participants are instructed to fill in a sleep diary for the last 7 days, documenting sleep timing, duration, waking up during the night, sleep quality, and daytime sleepiness on a continuous basis. This diary allows participants to self-report their sleep experiences, offering an informed view of their sleep patterns over the average of the last week also including any change in sleep-timing that would indicate a circadian effect of treatment.

In addition to the sleep diary, we employ the Pittsburgh Sleep Quality Index (PSQI) at baseline and day 42 of the study. The PSQI is a standardized measure that evaluates seven components of sleep quality, including subjective sleep quality, sleep latency, sleep duration, habitual sleep efficiency, sleep disturbances, use of sleeping medication, and daytime dysfunction.

### Adherence evaluation

Adherence to the device and treatment will be evaluated based on recorded statistics on device usage (in minutes per session). The eye-tracking system within the device will be utilized to quantify the active presence of the participant and gaze direction during each session. Adherence is considered high when the participant is present in front of the device for over 70% of the recommended session time (one hour).

### Exploratory analysis of electroencephalography data

Depression has been associated with various alterations in electroencephalographic (EEG) patterns. In terms of treatment, effective antidepressant therapies, including pharmacological interventions, psychotherapy, and neuromodulation techniques, are shown to cause EEG changes over time and in relation to recovery of faster gamma frequency oscillations [49]. The study will examine two different EEG conditions: resting state activity and SSVEPs responses to 40 Hz strobe light. EEG measurements will be collected at baseline and 6 weeks post-treatment visits to assess the effects of 40 Hz vs continuous light to the resting state activity and evoked responses of depressed patients. No hypothesis testing will be done regarding EEG measurements.

### Data collection

Identifiable data such as full name, address, and social security number will only be shared with key personnel and only for data collection from the electronic patient journal. All data sharing will be according to the European General Data Protection Regulation (GDPPR) and in agreement with permission from the Danish data protection agency. The REDCap system is used for collecting data during patient visits. The only data collected outside of the investigational site is the average usage time of the device, which is stored locally on the device and does not include any personal information. Depression severity measures (HAM-D_17_) will also be collected from the patients that dropped out.

Data collected: Age, gender, social security number, e-mail, phone number, inclusion date, diagnosis, sociodemographic data (residential status, work situation, somatic condition, prior and current depressive episode, smoking, eliciting factors for depressive episodes, suicidality history, alcohol, drug abuse), medical history, depression, mania, quality of life, sleep, side-effect, and suicidality ratings, treatment expectancy, blinding and treatment evaluation, concomitant medication.

### Statistical analysis plan

The analysis will be carried out using R software version 4.2.1 or higher (Free Software Foundation’s GNU General Public License). With a minimal clinically important difference of 3 points on the HAM-D_6_ scale, an expected standard deviation of 3.5, an alpha value of 0.05, and a power of 90%, and considering dropout, we will need a total of 60 patients to show the significance of the primary outcome. Based on the previous studies at Psychiatric Centre Copenhagen, dropout is expected to be below 25%, and we will attempt to administer the HAM-D_6_ (primary endpoint) for all the patients who drop out of the trial. An intention-to-treat population, including all randomized participants, will be used for analyses. The mean change from baseline to post-treatment at day 42 in HAM-D_6_ scores will be compared between treatment groups using a two-sample *t*-test. *T*-test assumptions of normality and equal variance will be checked. A mixed model repeated measures method will be used to analyze depression symptoms from baseline to measurements at day 7, day 21, day 42, and day 56. The Benjamini-Hochberg correction will be used for multiple comparisons. A *p*-value of less than 0.05 is considered a rejection of identity between groups.

## Ethics and dissemination

### Informed consent

Eligible subjects will be informed in detail about the study protocol by the study investigators. Oral and written informed consent will be obtained from each subject.

The study is approved by Danish Medicines Agency (case number 2023011518) and the Danish National Medical Research Ethics Committee (case number 2300521). Data will be handled according to the General Data Protection Regulation and the study is registered in the central registry for research in the Capital Region of Denmark (P-2022-922).

## Discussion

Implementing novel, effective long-term brain stimulation paradigms is crucial for the management of depression that does not respond to antidepressant medications and/or approved brain stimulation modalities or psychotherapy. By exploring the potential antidepressant, cognitive, and electrophysiological effects, the study aims to contribute to the development of more effective and personalized treatment options for MDD. The use of gamma-frequency brainwaves as a biomarker of interest holds promise in elucidating the underlying network mechanisms of MDD and providing potential therapeutic targets. However, certain limitations should be acknowledged. Firstly, the study might require larger sample size and duration to confirm the effects of light treatment on the patient population described above. Secondly, we expect to see a placebo effect of both types of light on depression symptoms and the primary endpoint. Lastly, the possible circadian effects of light treatment in both groups are not known and will affect study outcomes.

In conclusion, the study has the potential to contribute to the growing knowledge on gamma-frequency brainwaves and targeted interventions in MDD.

## Data Availability

After publication of the results, anonymized data can be made available from the principal investigator (K.M.) upon reasonable request.
